# Pouched Rats' Detection of Tuberculosis in Human Sputum: Comparison to Culturing and Polymerase Chain Reaction

**DOI:** 10.1155/2012/716989

**Published:** 2012-07-09

**Authors:** Amanda Mahoney, Bart J. Weetjens, Christophe Cox, Negussie Beyene, Klaus Reither, George Makingi, Maureen Jubitana, Rudovick Kazwala, Godfrey S. Mfinanga, Amos Kahwa, Amy Durgin, Alan Poling

**Affiliations:** ^1^Department of Psychology, Western Michigan University, Kalamazoo, MI 49008-5200, USA; ^2^Tuberculosis Research, Anti-Persoonsmijnen Ontmijnende Product Ontwikkeling (APOPO), Morogoro, Tanzania; ^3^Tuberculosis Research, Swiss Tropical and Public Health Institute, 4051 Basel, Switzerland; ^4^TB Research and Training Center, Ifakara Health Institute, Bagamoyo, Tanzania; ^5^Department of Veterinary Medicine, Sokoine University of Agriculture, Morogoro, Tanzania; ^6^Clinical Research Laboratory, National Institute for Medical Research, Dar es Salaam, Tanzania

## Abstract

*Setting*. Tanzania. *Objective*. To compare microscopy as conducted in direct observation of treatment, short course centers to pouched rats as detectors of *Mycobacterium tuberculosis*. *Design*. Ten pouched rats were trained to detect tuberculosis in sputum using operant conditioning techniques. The rats evaluated 910 samples previously evaluated by smear microscopy. All samples were also evaluated through culturing and multiplex polymerase chain reaction was performed on culture growths to classify the bacteria. *Results*. The patientwise sensitivity of microscopy was 58.0%, and the patient-wise specificity was 97.3%. Used as a group of 10 with a cutoff (defined as the number of rat indications to classify a sample as positive for *Mycobacterium tuberculosis*) of 1, the rats increased new case detection by 46.8% relative to microscopy alone. The average samplewise sensitivity of the individual rats was 68.4% (range 61.1–73.8%), and the mean specificity was 87.3% (range 84.7–90.3%). *Conclusion*. These results suggest that pouched rats are a valuable adjunct to, and may be a viable substitute for, sputum smear microscopy as a tuberculosis diagnostic in resource-poor countries.

## 1. Introduction

A major hurdle in combating tuberculosis (TB) is diagnosing the disease in resource-poor countries.Sputum smear microscopy, the technique typically used, is relatively slow and characteristically has high specificity but low sensitivity [1, 2]; therefore, the international medical community has prioritized developing a quick, accurate, and affordable alternative diagnostic. In an attempt to develop one, researchers recently have investigated the use of scent-detecting pouched rats (*Cricetomys gambianus*) as a TB diagnostic.An initial proof of principle investigation [3] revealed that pouched rats trained through operant conditioning procedures could detect TB in human sputum, and three subsequent studies, involving a total of over 20,000 patients, showed that using the rats in second-line screening of sputum samples initially screened by smear microscopy at direct observation of treatment—short course (DOTS) centers in Tanzania increased new case detections by 31.4% [4], 44% [5], and 42.8% [6].

These results are promising, but the accuracy of *Cricetomys* in detecting TB has not been extensively evaluated relative to an established reference standard. Culturing is considered the “gold standard” for TB detection [2], and Weetjens et al. [3] reported the results of a study in which two rats, Mandela and Kingston, evaluated 817 sputum samples also evaluated by culturing, which revealed 67 TB-positive samples. Sensitivity relative to culturing for both rats was 73.1%, while specificity was 97% and 97.8% for Mandela and Kingston, respectively. In an attempt to provide more comprehensive information regarding pouched rats' TB-detection accuracy relative to the best available and affordable method, this experiment evaluated 10 rats' performance compared to culture in combination with Multiplex PCR. 

## 2. Method

### 2.1. Subjects and Materials

Ten adult *Cricetomys* obtained from our breeding colony, 5 males and 5 females, evaluated all sputum samples. The animals were housed and maintained as detailed elsewhere [3, 7]. Ethical clearance to conduct the research was obtained from the Tanzanian National Institute for Medical Research. Some of the rats had been used in previous studies and all of the rats had been evaluating sputum samples for TB for at least one year. 

Testing was conducted in a chamber 205 cm long, 55 cm wide, and 55 cm high with clear plastic walls and ceiling and a stainless steel floor. Ten holes with sliding lids 2.5 cm in diameter were spaced equidistance apart along the centerline of the chamber floor's long axis. Pots containing sputum were placed beneath the holes for the rats to evaluate. Edible reinforcers (rewards), consisting of a mixture of mashed banana with ground rodent diet pellets, were delivered through a plastic syringe through feeding holes.

### 2.2. Collection of Sputum Samples

Sputum samples were collected weekly from eight DOTS centers in Dar es Salaam and Morogoro, Tanzania, using World Health Organization (WHO) recommended sputum containers. Direct smear microscopy after Ziehl-Neelsen staining was conducted at the DOTS centers prior to collection of the samples. Samples of less than 2 mL were excluded to ensure that there was sufficient volume for culturing and rat evaluation. In all, 910 samples from 456 patients (two from each of 454, one from each of two) were evaluated. Before evaluation by rats, an aliquot was taken from each sample for culture purposes, and then sterile phosphate buffered saline solution (5 mL) was added to each sputum sample and microorganisms were inactivated by heating the sample at 90°C in a water bath for 30 min [8]. The samples were then frozen at −20°C until the day of evaluation (up to seven days). Though there is some controversy surrounding the cellular impact of freezing and thawing sputum, past research suggests that samples may be kept frozen without significant alteration of cell quality or cell counts [9]. Furthermore, data collected internally suggest that the rats' performance is unaffected by the freezing procedures employed. Samples were thawed four times for the purpose of this study: once on the day of collection, once to take aliquots for culture, once to evaluate the sputum quantity and add buffer, and once on the day of evaluation by the rats. 

### 2.3. Rats' Evaluation of Samples

Prior to this study, the rats were trained to detect TB as detailed elsewhere [3, 7]. In the present study, each rat evaluated each sample twice, in a different order, across 13 sessions. In each session, 63 samples found negative by microscopy at DOTS centers and seven samples found positive were presented to the rats. The seven positive samples served as reinforcement opportunities to maintain the rats' indications while the remaining samples were categorized as “unknown”. During the sessions, the experimenter opened each hole in the cage as the rat passed over and sniffed. When the rat paused for 5 s (i.e., emitted an indicator response), the experimenter informed a data collector who then stated whether the sample was smear positive according to DOTS-center microscopy. If the rat made an indicator response above a smear-positive sample, the experimenter sounded a click and delivered food, after which the rat then moved to the next hole to continue evaluations. If the rat emitted an indicator response at a smear-negative sample, which is considered an unknown sample, the experimenter closed the hole but did not sound a click or present food. 

### 2.4. Data Analysis of Rat Results

Following evaluations, the rats' performance was assessed relative to the results of culture with *M. tuberculosis* Multiplex PCR. Sensitivity and specificity were calculated for the group of 10 rats, and thus the criterion for counting a rat-positive indication could be an indication on either or both sample presentations by one rat, ten rats, or any number of rats in between, which are referred to hereafter as cutoffs 1–10. At a cutoff of 3, for example, a sample was deemed rat positive if three or more rats indicated it; samples indicated by only 2, 1, or 0 rats were deemed negative. 

### 2.5. Culturing and PCR

Culturing was conducted in accordance with an established and recommended procedure for culturing sputum samples on Lowenstein-Jensen (LJ) solid media (WHO Guidelines on Standard Operating Procedures for Microbiology, Tuberculosis, WHO Regional Office for Southeast Asia, 2006). Decontaminated samples were inoculated onto different tubes of Lowenstein-Jensen solid media, one with pyruvate and the other with glycerol. The tubes were incubated at 37°C and inspected weekly for eight weeks. Media on which microbial growth was observed were scraped, stained by the ZN method, and analyzed by light microscopy. Each specimen which exhibited either AFB-positive culture material or characteristic bacterial growth was further analyzed by Multiplex PCR. 

In the first step of PCR [10], Multiplex PCR genus typing was conducted to identify species belonging to the *Mycobacterium* genus. This genotyping distinguished species belonging to the *Mycobacterium tuberculosis* complex (MTC), specifically *M. bovis*, *M. africanum*, *M. tuberculosis*, and *M. microti*, from nontuberculous mycobacteria (*M. avium*, *M. intracellulare*, and others). All bacterial suspensions or DNA extracts containing MTC were subjected to another PCR, deletion typing. This procedure differentiated bacteria that were *M. tuberculosis*, *M. bovis*, or *M*. *africanum*. 

## 3. Results 

All sputum samples were classified as positive or negative for *M. tuberculosis* by microscopy at the DOTS centers, culturing (with PCR as appropriate), and rats' evaluation. Culture-positive samples were those in which characteristic growth was stained with a Ziehl-Neelsen stain and the presence of acid-fast bacilli confirmed. A sample was further considered PCR-positive if, following amplification, the amplified nucleotide sequence for *M. tuberculosis* was detected (see Table 1 for these results). DOTS centers' microscopy identified 96 positive samples and 49 positive patients, and culture identified 162 positive samples and 104 positive patients. PCR identified 129 positive samples and 81 positive patients. DOTS-centers' microscopy found 86 of the PCR positive samples and 771 of the PCR negative samples. Thus, relative to PCR, sputum smear microscopy conducted at the DOTS centers yielded a sample-wise sensitivity of 66.7% and a specificity of 98.7%. 

Table 2 displays rat results relative to PCR at rat cutoffs 1–10. At a cutoff of 1, sample-wise sensitivity was 84.5% and specificity was 64%. As the cutoff increased, specificity characteristically improved while sensitivity worsened. At the largest cutoff, 10 (meaning all rats must indicate upon the sample to score it as rat-positive), sensitivity was 53.5% and specificity was 98.1%. At a cutoff of 7 sample-wise sensitivity was 65.1% and specificity was 93.3%. 

Although it is tenable to use 10 rats, using fewer animals would render the evaluation process faster and less expensive. To ascertain the effects of using fewer animals, 36 combinations of randomly selected rats were created from the present data set, 12 combinations of 4 rats, 12 combinations of 3 rats, and 12 combinations of 2 rats. Sample-wise sensitivity and specificity relative to culture/PCR were calculated for each rat combination (Table 3). Using four rats at a cutoff of one rat, average sensitivity was 79.9% (range 76.2–82.5%) and average specificity was 73.8% (range 71.2–75.4%). When only two rats were used, average sensitivity decreased to 74.9% (range 69–88.1%) while average specificity increased to 81.6% (range 78.1–85.6%). As the cutoff selected is increased, meaning more rat indications are required to count a sample as rat-positive, sensitivity decreases and specificity increases. For example, using four rats at a cutoff of four, sensitivity dropped to 57.8% (from 79.9% at a cutoff of 1) while specificity improved to 96.2% (from 73.8% at a cutoff of 1).

Table 4 shows patient-wise data comparing the sensitivity and specificity of DOTS centers' microscopy to the average for the 10 individual rats. Relative to combined Multiplex PCR and MTB/RIF results, DOTS centers' microscopy identified 47 of 81 positive patients and 407 of 409 negative patients. Therefore, at the patient-wise level, the sensitivity of microscopy at DOTS centers was 58% and the specificity was 99.5%. The rats correctly classified, on average, 57.1 positive and 329.1 negative patients and 104.2 positive and 675 negative samples. For the rats, the mean patient-wise sensitivity was 70.5% (99% confidence interval [CI] .68–.73) and mean specificity was 80.5% (95% CI .78–.83). Predictive values were calculated for patient-wise results of DOTS microscopy and the rats (Table 4). The positive predictive value (i.e., the probability that a patient with a positive test result really does have the condition of interest) was .96 (95% confidence interval [CI] .84–.99) for microscopy and .42 (95% CI .33–.50) for the rats. The negative predictive value (i.e., the probability that a patient with a negative test result really is free of the disease) was .92 (95% CI .89–.95) for microscopy and .93 (95% CI .89–.96) for the rats.

## 4. Discussion

In this study, 10 adult *Cricetomys* evaluated 910 sputum samples collected from patients suspected for tuberculosis. Weetjens et al. [3] previously reported that each of two pouched rats yielded a sensitivity of 73.1% relative to culturing, and their specificities were 97% and 97.8%. In the present study, which included Multiplex PCR, somewhat lower values were obtained where the mean individual sample-wise sensitivity of 10 rats relative to culture/PCR was 68.4%, and the mean specificity was 87.3%. Nonetheless, each rat's sensitivity exceeded that of ZN smear microscopy performed as part of routine TB screening at DOTS centers, although their specificity was lower. Because the rats can evaluate samples quickly, it is tenable to have several of them evaluate each sputum sample, and this has been done in studies examining their use in second-line screening of samples initially evaluated by ZN microscopy [4–6]. For example, Poling et al. [5] used a cutoff of 2 of 10 rats for identifying a sample as TB-positive. The present data suggest that this is a reasonable criterion in terms of balancing sensitivity and specificity, which were 81.4% and 75.7% when it was used in the present study. Similar values were obtained with cutoffs of 3 of 10 and 4 of 10. With a cutoff of 2, the rats as a group detected 66 of 81 patients found to be TB-positive by culturing/PCR, whereas DOTS centers' microscopy detected 47 of these patients. Therefore, had the rats been used in second-line screening as in prior studies [5, 6] and their results verified, they would have increased new-case detections by 40.4%. 

Results revealed 57 culture-negative and three culture-positive, but PCR-negative samples indicated by six or more rats. To clarify the status of these samples, an internal test was done on all of these plus 100 randomly-selected rat-negative samples using the GeneXpert MTB/RIF (Cephid, Sunnyvale, CA, USA) [11]. Analysis by the GeneXpert revealed 25 positive samples and 23 positive patients that had previously been classified as negative by culture or Multiplex PCR, bringing the combined Multiplex PCR and MTB/RIF positive samples to 154 and positive patients to 98. The test reclassified 18 of the 60 rat-positive samples and 7 of the 100 rat-negative samples. An analysis was conducted including these reclassified participants and revealed a patient-wise sensitivity of microscopy at DOTS centers of 48% and specificity of 98.3%. For the rats, patient-wise sensitivity was 67% (range 62.2–72.5%) while their mean specificity was 93.5% (range 91.1–95.3%). Due to the costs of the cartridges required for the GeneXpert, it was not possible to evaluate all samples and, to avoid a possible bias, these data were not incorporated into the main results. These additional data suggest that the specificity of the rats may be higher than that suggested by the comparison to culture and, to test this possibility these findings, a study is underway that will thoroughly evaluate the rats relative to MTB/RIF. 

In prior studies, a second microscopy was used to verify the status of DOTS-negative, rat-positive samples, but such confirmation is weak. The rats identify as positive a relatively high number of TB-negative samples; however, relying on microscopy alone allows a substantial number of patients with TB to go undetected. A better procedure would be to use the GeneXpert to confirm the status of rat-positive, smear-negative samples. Confirmation of samples in this way is likely to reveal a substantial number of TB-positive patients missed with the present procedure and thus slow the spread of transmission. This benefit would seemingly justify the financial cost of using the GeneXpert.

In addition to finding TB-positive patients overlooked by microscopy, the rats may potentially yield savings in time and cost. The rats are faster in evaluation than a lab technician, but require that the samples be transported and processed. These steps are completed with large batches of samples and it is important that future studies clearly demonstrate the cost-efficiency of these procedures relative to microscopy and investigate potential savings in costs and time. Prospectively, should the rats be called upon as a first-line screening tool, research on the MTB/RIF assay indicates that its high cost may limit its global utility [12], and the rats seem particularly well suited to work in conjunction with this technology to reduce costs. The rats screen samples quickly and, if used in areas with prevalence similar to that in which they have been tested, will reduce the number of patients in need of followup. The outcome of such a setup depends largely upon the number of rats used. Extrapolating from the current results, one could expect to recheck about 10% of samples if one rat is used and recheck about 45% of samples if 10 rats are used. The ideal number, as illustrated in Table 4, is probably between 2 and 4 rats as sensitivity remains relatively high while the false alarm rate improves with fewer rats. 

A significant limitation of the present study is that no clinical data were available for comparison. TB-positive patients evaluated by TB specialists are more likely to be identified than those diagnosed by smear results alone [13], and so a study is underway at APOPO that will incorporate clinical data. A second limitation is that the HIV status of patients was not available to us; therefore, the sensitivity and specificity of DOTS microscopy and the rats could not be compared in HIV-positive and HIV-negative patients. Further research in this area is planned to make the comparison and to evaluate the value of the rats in detecting TB in children. 

## 5. Conclusions

In this study, DOTS microscopy found 58% of the culture/Multiplex PCR positive patients, which is similar to results found in past studies [2, 11], compared to an average of 70.5% of positive patients found by individual rats. The results presented herein, combined with previously published operational data, demonstrate that the rats are faster than smear microscopy as commonly practiced and can identify more TB-positive patients. There is now substantial evidence that when used for second-line screening, *Cricetomys* can have a large positive impact on TB detection and public health in high-incidence areas, such as sub-Saharan Africa, although future research is necessary to refine training techniques to identify the applications for which the rats are best suited and to ascertain their per-case-detected cost relative to alternative diagnostics. 

## Figures and Tables

**Table 1:** Samples classified as *M. tuberculosis* and non-*M. tuberculosis* by Multiplex PCR.

	*M*. *tuberculosis*	Non *M. tuberculosis *
Total PCR+	129	13
ZN+ glycerol/pyruvate	(102/87)	(2/13)
^*α*^Rat+	109	10
Smear+ (DOTS)	86	7

^*α*^A sample was considered rat positive if at least one rat indicated.

**Table 2:** Sample-wise and patient-wise sensitivity and specificity at rat agreements cutoffs 1–10.

Cutoff	Samplewise		Patientwise	
	Sensitivity	Specificity	Sensitivity	Specificity
1	84.50	64.00	85.54	49.06
2	81.40	75.70	81.93	64.61
3	76.00	81.80	75.90	73.99
4	74.40	86.60	73.49	80.70
5	68.20	89.40	66.27	84.18
6	66.70	91.90	65.06	88.47
7	65.10	93.30	63.86	90.88
8	62.80	95.50	62.65	94.64
9	58.90	96.90	60.24	96.51
10	53.50	98.10	54.22	98.12

^*α*^Relative to Multiplex PCR.

**Table 3:** Average sensitivity and specificity for 12 groups of 4, 3, and 2 rats.

Cutoff	4 Rats		3 Rats		2 Rats	
	Sensitivity	Specificity	Sensitivity	Specificity	Sensitivity	Specificity
1	79.9	73.8	77.2	77.4	74.9	81.6
2	71.5	86.4	68.8	89.7	63.4	93.4
3	64.8	92.0	60.6	95.6		
4	57.8	96.2				

^*α*^Relative to culture/PCR.

**Table 4:** Patient-wise smear microscopy and average rat reference results.

	Sensitivity	Specificity	Pos predictive value	Neg predictive value
	Culture/PCR	No TB	Culture/PCR	No TB
^*α*^Smear				
Correct/total samples (%)	47/81 (58.0)	407/409 (99.5)	.96	.92
95% confidence interval (CI)	46.6–68.7	98–99.9	.84–.99	.89–.95

Rat (average of 10)				
Correct/total samples (%)	57.1/81 (70.5)	329.1/409 (80.5)	.42	.93
95% CI	68.2–72.8	78.4–82.6	.33–.50	.89–.96

^*α*^Smear microscopy conducted at DOTS centers.
